# Tunable Enzyme-Assisted Mineralization of Apatitic Calcium Phosphate by Homogeneous Catalysis

**DOI:** 10.3390/ijms24010043

**Published:** 2022-12-20

**Authors:** Brittany Foley, Clément Guibert, Mohamed Selmane, Alberto Mezzetti, Caroline Lefebvre, Karim El Kirat, Jessem Landoulsi

**Affiliations:** 1 Laboratoire de Biomécanique & Bioingénierie, Université de Technologie de Compiègne, CNRS, BP 20529, CEDEX, F-60205 Compiègne, France; 2 Laboratoire de Réactivité de Surface, Sorbonne Université, CNRS, F-75005 Paris, France; 3 Fédération de Chimie et Matériaux de Paris-Centre (FCMat) FR2482, F-75005 Paris, France; 4 Service d’Analyse Physico-Chimique, Université de Technologie de Compiègne, BP 20529, CEDEX, F-60205 Compiègne, France

**Keywords:** biomineralization, hydroxyapatite, X-ray powder diffraction, alkaline phosphatase, carbonate, fluoride

## Abstract

While it has long been mimicked by simple precipitation reactions under biologically relevant conditions, calcium phosphate biomineralization is a complex process, which is highly regulated by physicochemical factors and involves a variety of proteins and other biomolecules. Alkaline phosphatase (ALP), in particular, is a conductor of sorts, directly regulating the amount of orthophosphate ions available for mineralization. Herein, we explore enzyme-assisted mineralization in the homogeneous phase as a method for biomimetic mineralization and focus on how relevant ionic substitution types affect the obtained minerals. For this purpose, mineralization is performed over a range of enzyme substrate concentrations and fluoride concentrations at physiologically relevant conditions (pH 7.4, T = 37 °C). Refinement of X-ray diffraction data is used to study the crystallographic unit cell parameters for evidence of ionic substitution in the lattice, and infrared (IR) spectroscopy and X-ray photoelectron spectroscopy (XPS) are used for complementary information regarding the chemical composition of the minerals. The results show the formation of substituted hydroxyapatite (HAP) after 48 h mineralization in all conditions. Interestingly, an expansion of the crystalline unit cell with an increasing concentration of the enzyme substrate is observed, with only slight changes in the particle morphology. On the contrary, by increasing the amount of fluoride, while keeping the enzyme substrate concentration unchanged, a contraction of the crystalline unit cell and the formation of elongated, well-crystallized rods are observed. Complementary IR and XPS data indicate that these trends are explained by the incorporation of substituted ions, namely CO_3_^2−^ and F^−^, in the HAP lattice at different positions.

## 1. Introduction

The mineralized tissues of vertebrates are primarily composed of substituted apatites, sometimes called bioapatites, which consist of poorly crystalline apatitic calcium phosphate (CaP) mineral. This mineral phase differs from the stoichiometric hydroxyapatite (HAP, Ca_10_(PO_4_)_6_(OH)_2_) with the incorporation of various substituted ions (e.g., CO_3_^2−^, F^−^, Cl^−^, Na^+^, K^+^, Mg^2+^, etc.) assuming different positions throughout consequentially imperfect HAP-like lattices [1,2] but maintaining the characteristic hexagonal symmetry (space group P63/m, *a = b ≠ c, α = β =* 90° *≠ γ =* 120°).

Many in vitro methods have been explored for decades with the aim of synthesizing apatitic CaP minerals for biomedical applications through varying degrees of complexity [3]. These methods are mainly based on chemical precipitation carried out in different physicochemical conditions, sometimes mimicking physiological conditions, and possibly including (bio)macromolecules of interest [4].

In vivo, the CaP formation in hard mineralized tissues involves matrix vesicles (MVs), which are spherical microstructures identified as the initial sites where mineral forms [5,6]. In addition to their structural features, the main characteristic of MVs is related to their contents of specific proteins, selected to assist the nucleation and growth of CaP minerals, providing temporal and spatial control over the formation and maturation processes [7,8]. This is mainly achieved thanks to the compartmentalization induced by the liposome-like structure of MVs and their bounty of mineralizing enzymes [9]. In particular, tissue non-specific alkaline phosphatase (ALP, EC.3.1.3.1) is highly involved in the CaP mineralization process, as it generates orthophosphate ions (Pi) from a variety of organic phosphate molecules and inorganic pyrophosphate (PP_i_) [10,11,12,13]. Combined with the activity of other mineralizing enzymes, ALP contributes to the regulation of the PPi/Pi molar concentration ratio, which directly influences the HAP crystallization process [9,10,14,15].

Interestingly, ALP has recently been used for the development of biomimetic mineralization systems [16,17,18,19,20]. These studies have shown the possibility of modulating the mineralization process through a variety of parameters that influence the catalytic activity of the enzyme, including the concentration of the enzyme substrate [16], the temperature, and the pH of the mineralization solution [20].

In the interest of achieving further control over the growth and maturation of the mineral phase, particularly in terms of mineral morphology, mechanical properties, or chemical composition, we consider combining the previously listed controls with strategic ionic substitution. Substitutions throughout the HAP lattice can affect the size of the crystalline unit cell, strain, crystallite size, and morphology. Ionic substitutions, thus, significantly impact, among other factors, the physicochemical properties of the mineral phase which, in turn, determines its biological function [21]. Certain ionic substitutions affect the solubility of the mineral phase, an important characteristic that differentiates the mineral formed in different tissues. For example, some solubility of the mineral phase is necessary in bone in order to maintain the dynamics of bioresorption processes; the incorporation of carbonate ions in the lattice of bone mineral result in decreased crystallinity and increased solubility [22,23]. On the contrary, a lower solubility of the mineral phase is preferable in materials such as dental enamel, as this protective mineralized tissue must resist acid dissolution at the surface of the teeth; this is achieved by the substitution of *c*-axis channel hydroxyl ions by fluoride [24]. This ultimately yields a reinforced, less soluble, substituted HAP, which resists acid dissolution better than stoichiometric HAP [25]. The incorporation of fluoride into the lattice is, thus, widely explored for applications such as enamel repair and the design of enamel-like biomimetics, for which an acid-resistant mineral surface is sought to combat pathogenic dissolution in dental caries [26].

In this paper, we explore the biomimetic enzyme-assisted mineralization model in the homogenous phase and investigate how ions of biological interest, namely carbonate and fluoride, may be strategically incorporated into the mineral lattice. For this purpose, we characterize the CaP solids formed through an enzyme-assisted mineralization method in a homogenous phase with near-physiological conditions (pH = 7.4, T = 37 °C) over a 48 h incubation period. The obtained solids are analyzed by various techniques to characterize their composition and structure over multiple orders of magnitude; crystallographic information is determined from the refinement of XRPD data, and TEM provides complementary information about crystallinity and morphology. Chemical composition is probed using infrared spectroscopy in attenuated total reflection mode (IR-ATR) and X-ray photoelectron spectroscopy (XPS).

## 2. Results

### 2.1. Enzymatic Activity

**Figure 1:**
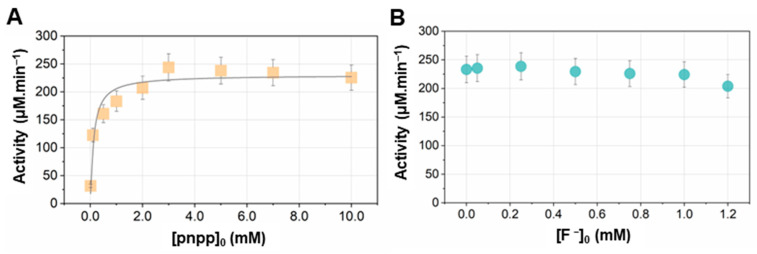
Evolution of the catalytic activity of ALP measured in Tris-HCl buffer solution (pH = 7.4; 37 °C) containing CaCl_2_ (11.4 mM) as a function of (**A**) the initial pnpp concentration [pnpp]_0_ or (**B**) the concentration of fluoride ions while [pnpp]_0_ = 7.0 mM. Error bars depict experimental uncertainty, evaluated at 10%. Experimental data (symbols) are the average values measured over three replicates and fitted (straight line, panel A) using the classical Michaelis–Menten equation.

The catalytic activity of ALP was studied in the homogenous phase with near-physiological conditions (pH = 7.4; T = 37 °C) and in the presence of calcium ions for all measurements (11.4 mM). The activity was measured at a fixed concentration of ALP (0.1 mg/mL) while varying the initial concentration of the substrate, pnpp, at values ranging from 0.010 to 10.0 mM. Results show that the activity increased with substrate concentration, as expected (Figure 1A). The activity–substrate concentration dependence is, however, difficult to fit in a reliable way. The kinetics of the ALP-catalyzed reaction were, indeed, broadly investigated following the classical Michaelis–Menten model, while an allosteric effect was reported [27,28]. An attempt to satisfactorily fit the activity–substrate concentration dependence was made using the classical Michaelis–Menten model (Figure 1A), yielding a constant value K_m_ = 0.119 mM and V_max_ = 230 µM.min^−1^ (adjusted R^2^ = 0.927). Note that the [S]_0_/v vs. [S]_0_ plot (v being the initial velocity of the enzyme-catalyzed reaction, i.e., the activity), showed a linear relationship, providing a K_m_ value equal to 0.116 mM (adjusted R^2^ = 0.998). 

At a substrate concentration [pnpp]_0_ = 7.0 mM, the enzymatic activity was shown to be slightly impacted by the presence of fluoride ions in the medium but variations remained well within the experimental uncertainties (Figure 1B).

### 2.2. XRPD

**Figure 2:**
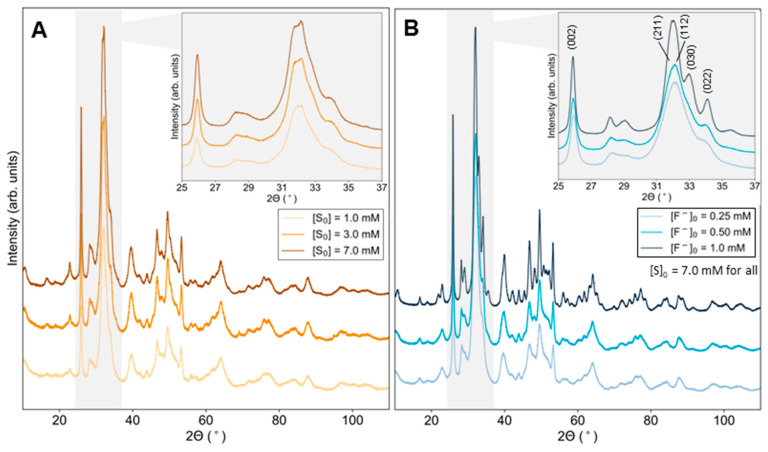
X-ray powder diffraction patterns for substituted HAP synthesized via homogeneous enzymatic catalysis at 37 °C, pH 7.4 (**A**) at variable concentrations of substrate, [S]_0_ = 1.0, 3.0 and 7.0 mM, or (**B**) at [S]_0_ = 7.0 mM and variable concentrations of fluoride ions, [F^−^]_0_ = 0.25, 0.50 and 1.0 mM.

Mineralized solids were analyzed by XRPD to determine their crystallographic structure and probe possible variations in the lattice with respect to mineralization conditions. All acquired diffractograms showed peaks that appeared consistent with stoichiometric or substituted HAP (P6_3_/m). Representative XRPD profiles for minerals formed at low, intermediate, and high substrate concentrations ([S]_0_ = 1.0, 3.0, and 7.0 mM, respectively) are displayed in Figure 2A. Prominent characteristic reflections include the sharpened (002) peak at approximately 2*θ* = 25.9°, typical for patterns of HAP crystals showing elongated growth in the *c*-direction, and the major peak at approximately 31.7° from (211) which overlapped with (112), (030), and (022) at roughly 32.2°, 32.9°, and 34.1°, respectively.

Representative XRPD profiles for minerals synthesized enzymatically with [S]_0_ = 7.0 mM and [F^−^]_0_ = 0.25, 0.50, and 1.2 mM are given in Figure 2B. Again, a sharpened (002) peak, which is suggestive of elongated growth along the crystallographic *c*-direction, was observed (indexed peaks indicated in Figure 2B, inset). The major peak for the equivalent reflections (121) and (211) overlaps with (112), (030), and (022) but was better resolved compared to samples that do not contain fluoride. In fact, peak resolution appeared to increase with increasing fluoride concentration, showing well-resolved peaks even in this overlapping region at higher concentrations ([F^−^]_0_ = 1.0 mM) (see insets in Figure 2A,B).

**Figure 3:**
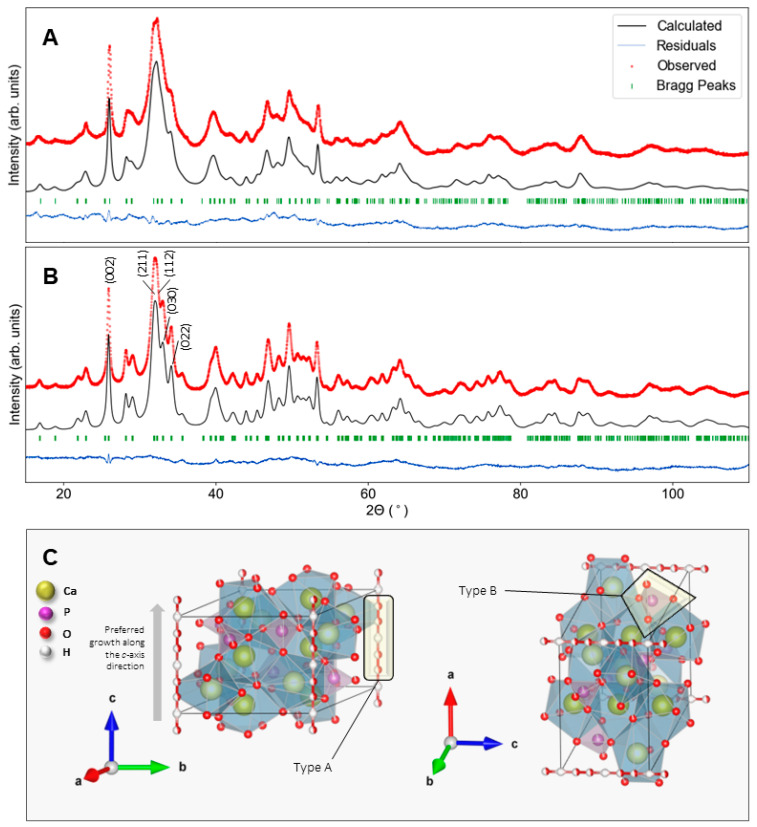
Derivative-difference minimization refinement (black) and residuals (blue) of measured X-ray powder diffraction patterns (red) for substituted HAP. Minerals were synthesized via homogeneous enzymatic catalysis at 37 °C, pH 7.4 using [S]_0_ = 7.0 mM and either (**A**) no added fluoride or (**B**) at [F^−^]_0_ = 1.2 mM. The calculated patterns were refined using (**A**) models for carbonate-substituted HAP (PDF4+ # 00-066-0147) and (**B**) fluoride- and carbonate-substituted HAP (PDF4+ # 04-015-8299), respectively (green). (**C**) Visualization of the crystallographic structure of HAP indicating sites of ionic substitution in the lattice.

Crystallographic lattice parameters were calculated by Rietveld refinement and using the DDM method, as described in 4.4. An example of the experimental versus calculated diffraction pattern generated from DDM refinement is plotted with residuals in Figure 3A,B for solids mineralized using [S]_0_ = 7.0 mM without the addition of fluoride and with [F^−^]_0_ = 1.2 mM, respectively. Bragg peaks are indexed for reference of constituent reflections in the chosen structural models, and important reflections are indicated in the callouts. A visualization of the crystallographic structure of one such model is provided in Figure 3C, with an arrow indicating the direction of the preferred elongated growth of HAP and substituted HAP in the *c*-direction. Regions of interest for ionic substitution are also highlighted therein; notably, these are the areas of substitution assumed by carbonate which can substitute either for hydroxyl ions aligned along the c-axis (called “Type A” substitution) or for tetrahedral phosphate (called “Type B” substitution).

**Figure 4:**
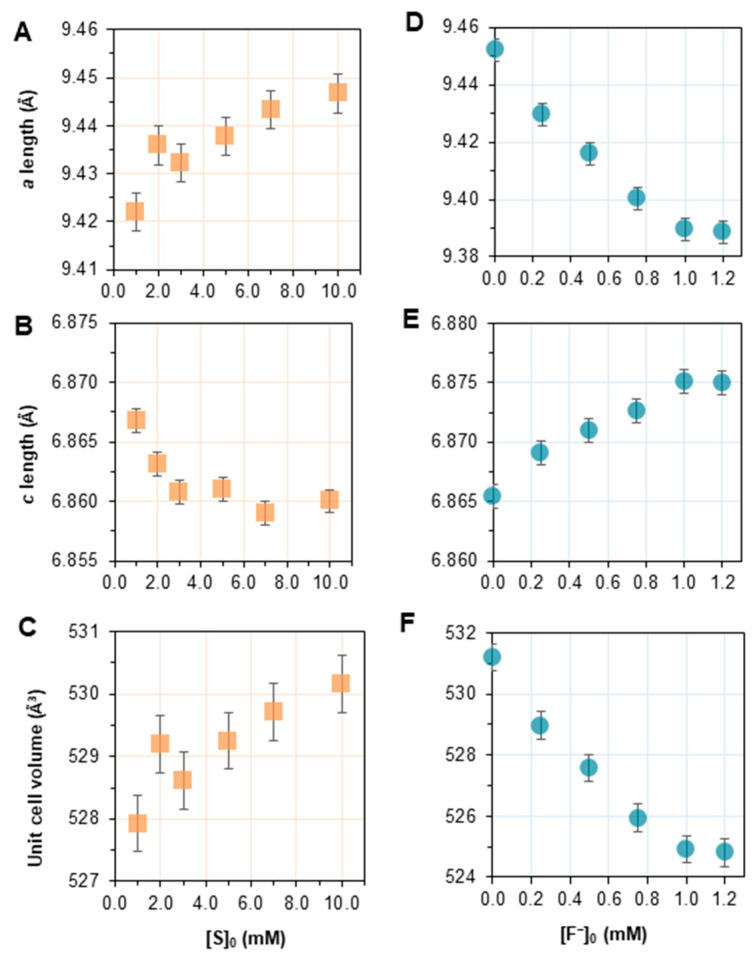
Comparative plots for unit cell dimensions of substituted HAP synthesized by homogeneous enzymatic catalysis (**A**–**C**) with variable substrate concentrations (orange squares) or (**D**–**F**) with [S]_0_ = 7.0 mM and variable fluoride concentrations (blue symbols). (**A**,**B**,**D**,**E**) Lengths of unit cell parameters were determined by refinement of X-ray powder diffraction data. (**C**,**F**) Unit cell volume was calculated from the lattice parameters based on the formula for a unit cell with hexagonal symmetry.

The *a* and *b* parameters, equivalent by hexagonal symmetry, and the *c* parameter are listed and visualized with respect to [S]_0_ in Table S1 (Supplementary Materials) and Figure 4A–C, respectively. The error is reported as the maximum observed difference between lattice parameters calculated by the Rietveld method and the DDM method. In Figure 4A, the *a* lattice parameters are plotted with respect to the initial overall substrate concentration, revealing a significant increase with [S]_0_, while an opposite trend was observed for the *c* lattice parameter (Figure 4B). In this case, an expansion of the unit cell volume was observed. Overall, the unit cell shows an increase in volume (calculated from the formula for a hexagonal unit cell using the calculated lattice parameters: $V=a^{2}c\times \sin\left(60{}^{\circ}\right)$) with increasing [S]_0_.

The *a* and *c* parameters calculated from the refinements are considered with respect to the amount of fluoride introduced in the mineralization solution in Table S1 (Supplementary Materials) and Figure 4D–F. The *a* length followed a roughly linear decrease with respect to [F^−^]_0_ up to 1.0 mM, at which point the parameter appears unchanged (Figure 4D). Meanwhile, the *c* length showed an opposite trend (Figure 4E), increasing as [F^−^]_0_ increases. The unit cell volume shows a contraction with increasing [F^−^]_0_ until [F^−^]_0_ = 1.0 and 1.2 mM, at which point the value remains unchanged, similar to what is observed for the *a* and *c* lengths (Figure 4F).

### 2.3. TEM

**Figure 5:**
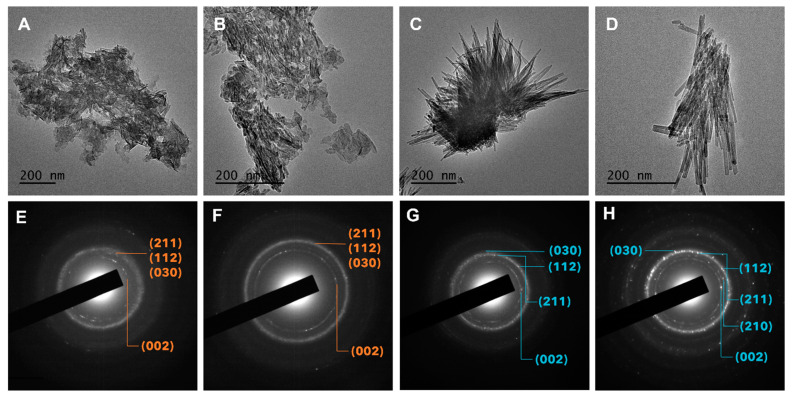
(**A**–**D**) Selected TEM micrographs and (**E**–**H**) SAED patterns recorded on substituted HAP synthesized via homogeneous enzymatic catalysis at 37 °C, pH 7.4 using (**A**,**E**) [S]_0_ = 1.0 mM, [F^−^]_0_ = 0; (**B**,**F**) [S]_0_ = 7.0 mM, [F^−^]_0_ = 0; (**C**,**G**) [S]_0_ = 7.0 mM, [F^−^]_0_ = 0.25 mM; (**D**,**H**) [S]_0_ = 7.0 mM, [F^−^]_0_ = 1.0 mM.

Some morphological variability with respect to different mineralization conditions was observed by TEM. Aggregation/agglomeration was observed in all samples; however, qualitative differences in particle shape and size may be observed between extreme conditions. Mineralization without the addition of fluoride at [S]_0_ = 1.0 mM yielded a mixture of small platelets and short needles whereas mineralization without fluoride using [S]_0_ = 7.0 yielded nanometer-scale, platelet-like solids (Figure 5A,B, respectively). The addition of fluoride resulted in a substantial change in morphology from platelets to highly regular elongated rods (Figure 5C,D). Minerals synthesized using [F^−^]_0_ = 0.25 mM appeared as fine rods that tended to aggregate together and reached several hundred nanometers in length (Figure 5C). Minerals synthesized using [F^−^]_0_ = 1.0 mM had a similar morphology with elongated rods, which appeared to show slightly higher width overall and ranged from fifty to several hundred nanometers in length (Figure 5D). SAED patterns showed diffraction rings (002), (030) and (112) and (211), typical of HAP (Figure 5E,F), which appeared more distinctly on mineral samples synthesized using [F^−^]_0_ = 0.25 (Figure 5G) and 1.0 mM (Figure 5H).

### 2.4. IR-ATR

**Figure 6:**
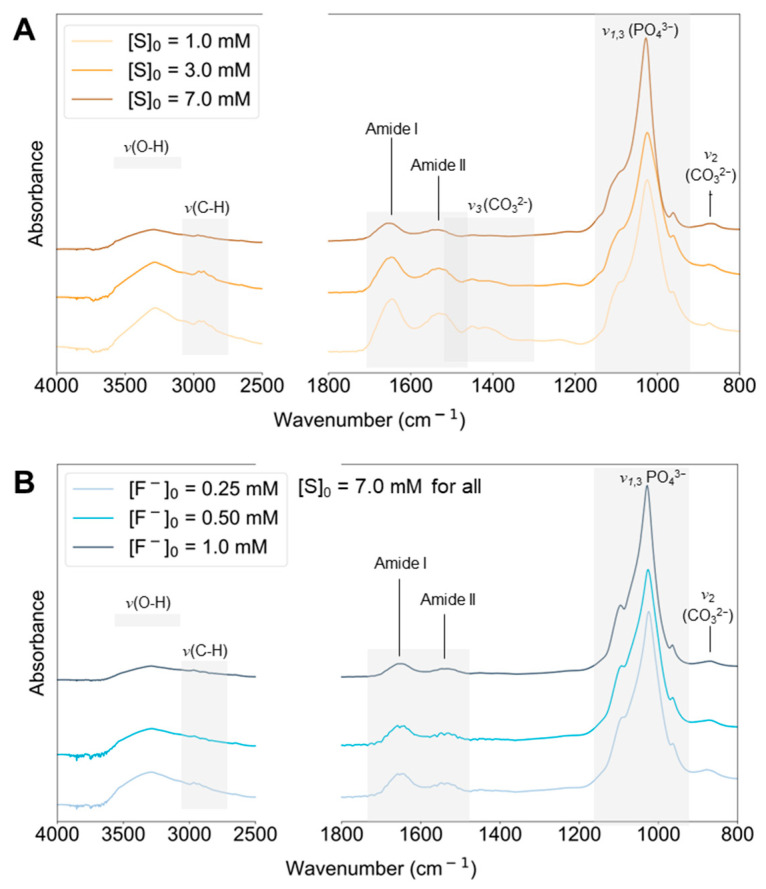
Selected IR-ATR spectra of substituted HAP synthesized by homogeneous enzymatic catalysis (**A**) at variable substrate concentrations, [S]_0_ = 1.0, 3.0 and 7.0 mM, or (**B**) at [S]_0_ = 7.0 mM and variable fluoride concentrations [F^−^]_0_ = 0.25, 0.50 and 1.0 mM.

The chemical composition of the different minerals was analyzed using IR-ATR. Typical IR spectra recorded on selected samples, namely enzymatically synthesized substituted HAP at [S]_0_ = 1.0, 3.0, and 7.0 mM without fluoride ions, or at [S]_0_ = 7.0 mM with [F^−^]_0_ = 0.25, 0.50, and 1.2 mM, are depicted in Figure 6A,B, respectively.

In all spectra, the *ν*_1_,*v*_3_ phosphate band contributions appear most prominent, spanning from 900–1200 cm^−1^ and resulting from the phosphate groups in the mineral phase (Figure 6A).

Amide bands were also observed in all spectra. Two broad components, visibly well-resolved and centered at approximately 1651 and 1540 cm^−1^, are assigned to the amide I and amide II bands, respectively. These bands indicate the presence of adsorbed enzymes on the mineral solids. A broad band assigned to O-H stretching is observed over the characteristic range from approximately 3200–3550 cm^−1^.

The presence of carbonate in the mineral phase is observed by its *v*_2_ and *v*_3_ vibrational modes. The *v*_3_ band, which overlaps with the amide band region, is poorly resolved in most spectra. However, all spectra also feature a weaker but better-resolved band spanning from approximately 840–890 cm^−1^, a region characteristic of bands for the *v*_2_ vibrational mode of carbonate. The IR vibrational features and band assignments are detailed in Table S2 (Supplementary Materials).

## 3. Discussion

### 3.1. Effect of the Substrate Concentration, [S]_0_

The evolution of lattice parameters with respect to the substrate concentration, [S]_0_, given in Figure 4A,B, revealed that the *a* parameter generally increases with [S]_0_, while the *c* parameter slightly decreases. By comparing the observed lattice parameters to those of stoichiometric HAP (*a =* 9.41844(3) Å, *c =* 6.88374(3) Å for PDF4+ # 00-009-0432), it is shown that a slightly higher *a* parameter and a lower *c* parameter are already observed even at the lowest [S]_0_ value, 1.0 mM (Figure 4A,B), suggesting that an ionic substitution may be involved at all of the studied conditions.

The unit cell volume showed a positive trend with [S]_0_ overall, with the increasing *a* parameter resulting in an expansion of the unit cell (Figure 4C). Indeed, various ionic species are known visitors to the classical HAP lattice, including Mg^2+^, Na^+^, CO_3_^2−^, F^−^, Cl^−^, etc. [2]. However, in the present study, only F^−^, CO_3_^2−^, Na^+^, and Cl^−^ were introduced or could be present in the mineralization solutions; fluoride is introduced systematically, sodium and chloride are introduced with pH adjustment and as reagent counterions, while carbonate may be incorporated as a consequence of the aqueous dissolution of atmospheric CO_2_. Neither sodium nor chloride was detected by XPS. In the absence of fluoride, carbonate, which is evidenced by infrared spectra, is eligible for consideration as a substituting ion.

The crystallographic consequences of CO_3_^2−^ incorporation into the HAP lattice were thoroughly investigated throughout the literature [29,30,31]. It is known that carbonate can occupy either or both of two positions: that of a hydroxyl group along the *c*-axis channel or that of a tetrahedral phosphate group [29]. The substitution mode is classified as either Type A or Type B, respectively, or Type AB if substitution is observed in both locations simultaneously. The presence of CO_3_^2−^ may also be due to the presence of adsorbed carbonate species (also called labile carbonate) but their presence is not expected to impact significantly the lattice parameters of the HAP solid. Substitution of HO^-^ by the bulkier CO_3_^2−^ (Type A) reportedly results in dilation of the *c*-axis channel and, thus, an increase in the *a* lattice parameter [29,32,33,34,35]; a slight contraction of the *c* parameter is also noted as the carbonate is oriented perpendicular to the channel. Conversely, the substitution of PO_4_^3−^ by the substantially smaller carbonate ion (Type B) results in the *a* lattice parameter contracting and the *c* parameter consequentially expanding [29,35,36]. The trend of lattice parameter changes observed herein (Figure 4A–C) suggests a variation of the amount of Type A substitution.

**Figure 7:**
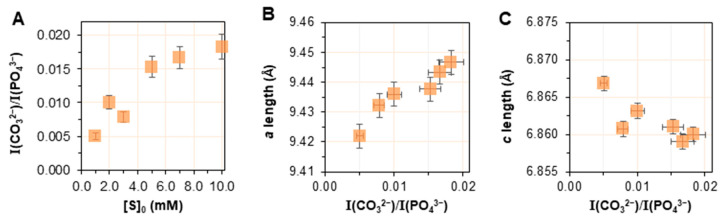
(**A**) Intensity of *v*_2_(CO_3_^2−^) band divided by the intensity of *v*_1,3_(PO_4_^3−^), I(CO_3_^2−^)/I(PO_4_^3−^), plotted as a function of [S]_0_; (**B**,**C**) *a* and *c* lattice parameters plotted as a function of the I(CO_3_^2−^)/I(PO_4_^3−^) ratio.

The integrated intensities of bands (i.e., the area of the band) due to carbonate (*v*_2_ band region, approximately at 820–915 cm^−1^) and phosphate (*v*_1_, *v*_3_ band region, approximately 910–1200 cm^−1^) groups were used to provide a relative estimate of the amount of carbonate in the mineralized solids. The use of this ratio has to be considered with appropriate caution as a broad hydrogen phosphate band can overlap with the *v*_2_ carbonate mode at approximately 870 cm^−1^, complicating the interpretation [37]. Figure 7A shows that the I(CO_3_^2−^)/I(PO_4_^3−^) appreciably increased as a function of the substrate concentration, [S]_0_, suggesting an enrichment of the mineral with carbonate species.

Figure 7B,C provide further information considering the incorporation of carbonate ions into the lattice, showing that the *a* length increases with respect to the relative carbonate content and the *c* length follows a less regular trend but decreases overall. These trends are consistent with the incorporation of carbonate in the lattice with mainly a Type A substitution mode. Support for this hypothesis is provided by the discrimination of Type A, Type B, or labile carbonate species based on the decomposition of ν_2_(CO_3_^2−^) band regions, as described in Figure S3 (Supplementary Materials). It is shown, indeed, that the component at about 894 cm^−1^, due to Type A carbonate, appeared at [S]_0_ = 3 mM, and then its intensity increased markedly with increasing [S]_0_.

The above findings clearly indicate that the variation of the substrate concentration, [S]_0_, leads to the incorporation of variable amounts of carbonate in the solid. However, this seems to not correlate with the enzymatic activity. Indeed, the activity increases up to [S]_0_ = 2.0 mM and then reaches a plateau at [S]_0_ values ranging from 2.0 to 10.0 mM (Figure 1A) whereas, in this concentration range, the incorporation of carbonate as substituted ions continues to grow (Figure 7A).

The enzymatic activity corresponds to the initial rate of phosphate production measured within the first seconds of the reaction. Accordingly, these results suggest that the amount of substituted carbonate in the lattice of HAP formed after 48 h mineralization is not dictated by initial enzymatic reaction kinetics. Instead, the observed effect may be explained by considering the total amount of phosphate ions generated in situ by the enzymes and accumulated in the mineralization medium during 48 h. The relevance of this parameter is supported by mineralization tests performed without enzymes via chemical precipitation under similar conditions as for the enzyme-assisted mineralization experiments, except that inorganic phosphate (Pi) was introduced instead of the enzyme–substrate (Method S1, Supplementary Materials). Indeed, these experiments show an evolution of the crystallographic structure similar to that observed for enzymatic mineralization (Figures S4–S6, Table S3, Supplementary Materials). If according to a previous study, the first stages of the mineralization were found to be significantly different between enzyme-assisted mineralization and chemical precipitation processes [16], both methods seem to lead to the same final materials after 48 regarding the mineral structure.

It is worth reminding that carbonate species are present in the mineralization solution due to the aqueous dissolution of atmospheric CO_2_ gas, and its amount mainly depends on the pH of the medium. This explains the incorporation of carbonate ions in the HAP lattice in all conditions, i.e., regardless of [S]_0_ and [F]_0_ values. The mechanism by which carbonates are incorporated in the HAP lattice in higher proportions when increasing either [S]_0_ (enzyme-assisted mineralization) or [Pi]_0_ (chemical mineralization) is intricate. This question deserves further investigation and, in particular, the characterization of the composition of intermediate products and the study of the nature of the phase transformation processes.

However, assuming that the substitution of carbonate species occurs at the mineral/solution interface during the growth of CaP particles, the increase in the concentration of particles is expected to favor ion exchanges between the mineral surface and the solution. Accordingly, when the concentration of CaP particles in the mineralization solution increases, due to an increase in either [S]_0_ or [Pi]_0_, the incorporation of Type A carbonate ions in the CaP lattice is favored. It then leads to a significant expansion of the unit cell volume (Figure S6, Supplementary Materials).

### 3.2. Effect of the Fluoride Concentration, [F^−^]_0_

The amount of fluoride introduced in the mineralization solution at [S]_0_ = 7 mM also yielded crystallographic changes. While increasing the amount of substrate caused an increase in the unit cell volume (Figure 4C), increasing the amount of fluoride up to [F^−^]_0_ = 1 mM resulted in a contraction of the unit cell volume (Figure 4F) with a decrease in the *a* lattice parameter and an increase in the *c* parameter (Figure 4D,E).

**Figure 8:**
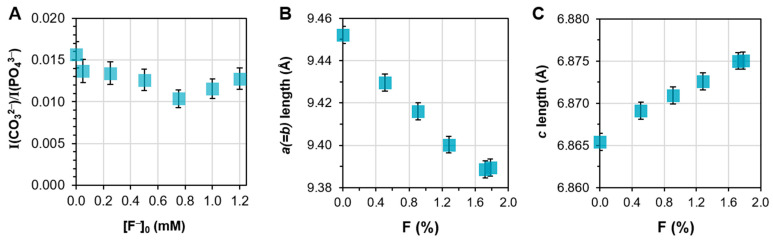
(**A**) Intensity of *v*_2_(CO_3_^2−^) band divided by the intensity of *v*_1,3_(PO_4_^3−^), I(CO_3_^2−^)/I(PO_4_^3−^), plotted with respect to [F^−^]_0_ initially introduced in the mineralization solution; (**B**,**C**) *a* and *c* lattice parameters plotted as a function of the molar concentration of fluoride as determined by XPS.

In this case, ionic substitution by both carbonates and fluorides was considered to explain the parameters observed. Without any added fluoride, the observed *a* length is still greater than that expected for stoichiometric HAP (9.4522(4) Å compared to the expected ~9.418 Å, PDF4+ #00-009-0342), which can be explained by CO_3_^2−^ substitution, as described above. Considering I(CO_3_^2−^)/I(PO_4_^3−^) in the range of [F^−^]_0_ investigated (Figure 8A), the carbonate content appears relatively stable compared to the range observed in Figure 7A. The variations remain, indeed, within the uncertainties due to calculations over the studied range of [F^−^]_0_. This suggests fluoride substitution in the lattice instead for the variation of lattice parameters observed in the variable-[F^−^]_0_ series.

Fluoride substitution in the HAP lattice was, indeed, explored at length in the literature, and is known to occur for hydroxyl groups occupying the *c*-axis positions. The substitution of fluoride in the lattice is characterized by a contraction of the *a* lattice parameter, as electronegative fluoride are expected to interact with surrounding calcium ions and occupy a position at their center, slightly offset from the position of native hydroxyl ions [25,38,39,40], while the evolution of the *c* length does not follow a clear trend. Figure 8B,C show that the *a* length decreased, while the *c* length increased significantly with respect to the molar concentration of fluoride in the mineral, as measured by XPS, which showed a linear relationship with [F^−^]_0_ up to 1.0 mM (Figure S7, Supplementary Materials). These trends clearly indicate that fluoride substitution is the cause of the observed crystallographic changes. While such an effect on the *c* length is not consistently reported for the substitution of fluoride, this may be described as a result of a reorganization of carbonate within the HAP lattice, as fluoride ions preferentially substitute in the *c*-axis position.

## 4. Materials and Methods

### 4.1. Chemicals

All solutions were prepared using ultrapure water (MilliQ, Millipore, France). For use in synthesis by enzymatic catalysis, alkaline phosphatase isolated from bovine intestinal mucosa (ALP, lyophilized powder, ≥10 DEA units/mg solid), glycerol phosphate disodium salt hydrate (S, isomeric mixture, typically 50% β-isomer and 50% rac-α-isomer), calcium chloride (CaCl_2_, anhydrous, ≥99%), and sodium fluoride (NaF, BioXtra, ≥99%) were all sourced from Sigma-Aldrich (France). All solutions were prepared in 10 mM Tris(hydroxymethyl)aminomethane hydrochloride (Tris-HCl) buffer, pH 7.4, made from Trizma^®^ base (minimum 99.9% titration) and 1 N hydrochloric acid (HCl). For measurement of enzymatic activity, 4-nitrophenol (pnp, ReagentPlus^®^ ≥ 99%) and 4-nitrophenyl phosphate disodium salt hexahydrate (pnpp, ≥99%) were sourced from Sigma-Aldrich. For pH adjustment, hydrochloric acid (HCl, 37%) from VWR and sodium hydroxide (NaOH, anhydrous pellets) from Carlo Erba Reagents were used.

### 4.2. Mineralization Procedure

Enzyme-assisted mineralization was performed using ALP and glycerol phosphate disodium salt hydrate as substrate (S). To explore the effect of enzymatic activity on mineral formation, mineralization solutions were prepared using a range of initial overall concentrations of substrate after having added and mixed all other components: [S]_0_ = 1.0, 2.0, 3.0, 5.0, 7.0, and 10.0 mM. To explore the effect of fluoride concentration on mineral formation, mineralization solutions were then prepared with a fixed [S]_0_ of 7.0 mM and a variable amount of NaF: [F^−^]_0_ = 0, 0.25, 0.50, 0.75, 1.0, and 1.2 mM. The enzyme–substrate was solubilized in buffer, and then CaCl_2_ was added from 1.0 M stock for an overall initial concentration of 11.4 mM. In solutions containing fluoride, NaF was added from 0.10 M solution in 10 mM Tris-HCl buffer. Mineralization was initiated with the addition of ALP, which was added from concentrated stock solutions with stirring for a final concentration of 0.1 mg/mL. All the mineralization solutions were incubated at 37 °C with gentle agitation on an orbital shaker for 48 h. After incubation, solutions were centrifuged (1244 relative centrifugal force, 10 min, room temperature) and supernatant was decanted. The remaining pellets were washed with equivalent volumes of MilliQ water, centrifuged, decanted thrice, and then dried at 60 °C for 16 h.

### 4.3. Enzymatic Activity Assay

Enzymatic activity of ALP in solution was measured at 37 °C using pnpp as substrate by monitoring the production of pnp in Tris-HCl buffer with 11.4 mM CaCl_2_. The pnp concentration was determined by measuring the absorbance at 410 nm using a UV–Vis spectrophotometer (Biochrom Libra S60PC, Cambridge, UK). At this wavelength, the other reagents do not interfere with the absorbance measurements. A calibration curve was preliminarily established relating the absorbance measured at 410 nm to pnp concentration in the solution (Figure S1, Supplementary Materials).

Two series of experiments evaluating enzymatic activity were performed using the pnpp colorimetric assay. In one experiment, the ALP activity was measured with respect to variable substrate (pnpp) concentrations. In the other, the initial concentration of pnpp was held constant at 7.0 mM and activity was measured in the presence of increasing amounts of fluoride ions.

Samples for each condition were prepared and measured in triplicate. The initial enzymatic activity of ALP for a given condition was then defined as the slope of the initial linear portion of the curve of pnp formed (µM) vs. time (min) (Figure S2, Supplementary Materials).

### 4.4. X-ray Powder Diffraction (XRD)

Crystallographic characterization of each sample was performed using X-ray powder diffraction. All solids obtained from the different mineralization conditions were isolated from solution by centrifugation, oven-dried and then micronized prior to characterization by grinding with mortar and pestle. Measurements were performed using a Bruker Discover D8 X-ray powder diffractometer (Bruker, Karlsruhe, Germany) with Bragg–Brentano geometry and 280.0 mm goniometer radius. Specifically, a copper X-ray anode (λ Cu K_α1_ = 1.54056 Å, K_α2_ = 1.54439 Å) was used at 40 kV, 30 mA. Samples were measured in coupled two θ/θ scanning mode from 2θ = 9–110° with a step size of 0.020° at 7.00 s per step and per channel using a 2A LYNXEYE XE-T detector in 1D mode with 192 channels. Experiments were conducted at ambient pressure and temperature.

Profile matching was first performed using the Bruker EVA.DIFFRAC analysis suite [41]. Rietveld refinement of diffraction data was performed using the FullProf [42] software suite and code together with the DDMsuite Derivative Difference Minimization program [43]. Peak indexing and data visualization were performed using the WinPLOTR program within FullProf [44,45] and Jupyter Notebook (v6.3.0) [46], respectively. For samples without fluoride, refinements were performed using both a classical HAP model (PDF4+ # 00-009-0432) and a carbonate-substituted HAP model each with hexagonal (P63/m) symmetry (PDF4+ # 00-066-0147), and the carbonate-substituted model was ultimately selected for as the more appropriate model. For samples containing fluoride, a calcium-deficient, fluoride- and carbonate-substituted model with hexagonal (P63/m) symmetry (PDF4+ # 04-15-8299) showed good agreement between experimental and calculated diffraction profiles.

Refinement was performed over the range of 2θ = 15–110°. It was initially performed with the derivative difference minimization technique using a convolution interval width of 2θ = 2° and 10-point weight baseline curve. The scale factor was refined first, followed by the sample displacement, *a* and *c* unit cell parameters, isotropic broadening factor *Z*, peak shape parameter, and anisotropic size broadening coefficients. The standard LaB_6_ was used for acquisition of the instrumental resolution function and aided the fitting of the Caglioti parameters U, V, and W, for a pseudo-Voigt profile. Refinement was additionally performed using the Rietveld refinement technique with the application of a Thompson–Cox–Hastings pseudo-Voigt profile. Initially, scale factor and sample displacement were refined, followed by the gradual refinement of a six-coefficient polynomial background function. An LaB_6_ standard was fit for the determination of instrumental broadening by first manually selecting suitable Lorentzian isotropic peak broadening parameters, X and Y, and then refining both with the isotropic peak broadening parameters (U_s_, Z_s_, X_s_, and Y_s_). Peak shape parameters were then refined to account for experimental contributions to peak broadening. Considerable peak broadening in the samples necessitated the manual selection of appropriate Lorentzian isotropic peak broadening parameters (X_s_, Y_s_) to begin refinement of lattice parameters. Isotropic peak broadening parameters and anisotropic size broadening coefficients were then refined, followed by anisotropic temperature factors and preferred orientation.

Lattice parameters for each sample were determined using both the Rietveld refinement (herein) as well as using the derivative difference minimization technique. The lattice parameter values from the appropriate ICDD model were used as starting parameters. For determination of the error in lattice parameter values obtained by refinement, DDM fitting and whole-pattern fitting over the same range values were compared and the error is provided as the maximum difference obtained between the two, having slightly surpassed the estimated standard error from the Rietveld refinement alone and having achieved an improved background and fit by DDM compared to Rietveld.

### 4.5. Infrared Spectroscopy in Attenuated Total Reflection (IR-ATR) 

Powder samples were analyzed by infrared spectroscopy for characterization of bulk chemical composition. Samples were analyzed using a Bruker VERTEX 80 FTIR spectrometer (Bruker Optik GmbH, Ettlingen, Germany) in attenuated total reflection (ATR) mode with a Bruker A225 mono-reflection diamond ATR device and liquid nitrogen cooled LN-MCT detector. Measurements were executed using the Opus 7.8 software (Opus, Version *7.8.44*. Bruker Optik GmbH: Ettlingen, Germany). Measurements were collected over a spectral range of 800–4000 cm^−1^ with 160 kHz scanning mirror speed. Background was measured in ambient air over 1000 scans, and experimental spectra were obtained using 250 scans per measurement, with powders measured in triplicate for each sample condition.

### 4.6. X-ray Photoelectron Spectroscopy (XPS)

XPS analyses were performed on powder samples fixed on indium foil using an ESCA+ spectrometer (Omicron Nano-Technology) with a monochromatic aluminum X-ray source (14 kV power, 20 mA current) and MCD 128 channeltron detector. The charge stabilization was ensured using a CN10 device operating at 5.0 µA emission current (0.30 eV beam energy). Measurements were performed in the sweep mode, yielding an analyzed area with an average diameter of 1 mm. A pass energy of 20 eV was used for narrow scans. The pressure in the analysis chamber was around 10^−10^ Torr. The photoelectron collection angle, θ, between the normal to the sample surface and the analyzer axis was 45°. The following sequence of spectra was recorded: survey spectrum, C 1s, N 1s, O 1s, F 1s, Na 1s, P 2p, Cl 2p, Ca 2p and C 1s again to check for charge stability during the analysis and the absence of sample degradation due to the exposure to X-rays. XPS peaks were referenced to the C 1s peak due to carbon bound to only carbon and hydrogen at 284.8 eV. The data treatment was performed with CasaXPS software (Casa Software Ltd., Teignmouth, UK) [47].

### 4.7. Transmission Electron Microscopy (TEM)

TEM images were recorded using a JEOL JEM-2100F instrument (Japan) equipped with a CCD camera and operating at an acceleration voltage of 200 kV. The crystallinity of HAP particles was evaluated using selected area electron diffraction (SAED) mode and high-resolution transmission electron microscopy (HRTEM). The obtained solids, collected as described above, were dispersed in ultrapure water, and a drop (10 µL) was deposited onto a copper-mesh grid coated with an amorphous carbon film, and then allowed to air-dry before imaging.

## 5. Conclusions

In summary, we showed here that the enzyme-assisted mineralization of CaP is a straightforward yet relevant way to modulate the process of ion substitution within the mineral lattice. Investigating this process required a thorough characterization of the mineral solid in terms of structure, morphology and composition, and was achieved using XRPD, including the refinement of X-ray diffraction data, IR and XPS. The present study reveals that the in situ generation of phosphate ions by free enzymes in solution leads to the formation of crystalline HAP involving variable substitution degrees of carbonate, present due to the dissolution of atmospheric CO_2_, and fluoride ions, deliberately introduced in the medium. The results showed a clear dependence between the concentration of the enzyme substrate, [S]_0_, and the lattice parameters which suggests the incorporation of carbonate ions. The unit cell expansion observed is characteristic of Type A substitution for hydroxyl ions along the c-axis channel. The addition of fluoride ions in the mineralization solution also showed crystallographic changes due to the incorporation of these ions, in addition to carbonates, within the HAP lattice.

The investigations described in this paper provide preliminary guidelines for the synthesis of biologically relevant HAP with tunable properties and may be extended to other inorganic ions, and even biomolecules, which modulate the nucleation and growth steps during the mineralization.

## Supplementary Materials

The following Supplementary Materials can be downloaded at: https://www.mdpi.com/article/10.3390/ijms24010043/s1.
