# Supplementary material for: Tunable Enzyme-Assisted Mineralization of Apatitic Calcium Phosphate by Homogeneous Catalysis
Source: Int J Mol Sci. 2022 Dec 20;24(1):43. doi: 10.3390/ijms24010043 (PMC9820226; doi:10.3390/ijms24010043)
Supplement: Supplementary file 1 [file ijms-24-00043-s001.zip › ijms-2020218-supplementary.pdf]

Tunable Enzyme-Assisted Mineralization of Apatitic Calcium Phosphate by Homogeneous Catalysis

Supplementary Materials

Brittany Foley [1,2], Clément Guibert [2], Mohamed Selmane [3], Alberto Mezzetti [2], Caroline Lefebvre [4], Karim El Kirat [1] and Jessem Landoulsi [1,2,*]

Table of Contents

Figure S1 – Calibration curve for measurement of enzymatic activity

Figure S2 – Initial enzymatic activity measured with respect to variable amount of enzymatic substrate introduced (A) and variable amount of fluoride introduced (B)

Figure S3 – Curve-fitting of the v_2 CO_3^{2-} bands of infrared spectra for substituted hydroxyapatite prepared via enzyme-assisted mineralization with variable $[S]_0$

Figure S4 – Selected X-ray powder diffraction patterns of substituted hydroxyapatite synthesized via chemical precipitation at 37°C, pH 7.4 using variable concentration of P_i

Figure S5 – Selected ATR-FTIR spectra of substituted hydroxyapatite synthesized by direct precipitation in solution using a range of initial concentration*s* of P_i

Figure S6 – Comparative plots for unit cell dimensions and relative carbonate content of substituted HAP synthesized by direct precipitation and enzyme-assisted mineralization

Figure S7 – Plot of fluoride content detected by XPS versus fluoride introduced during synthesis, $[F^-]_0$

Table S1 – Table of crystallographic lattice parameters determined by Rietveld refinement of X-ray powder diffraction data for samples synthesized by enzymatic catalysis with and without fluoride

Table S2 – Table of IR absorption band assignments

Table S3 – Table of crystallographic lattice parameters determined by Rietveld refinement of X-ray powder diffraction data for samples synthesized by chemical mineralization via direct precipitation in solution

Method S1 – Mineralization procedure for chemical mineralization

Figure S1

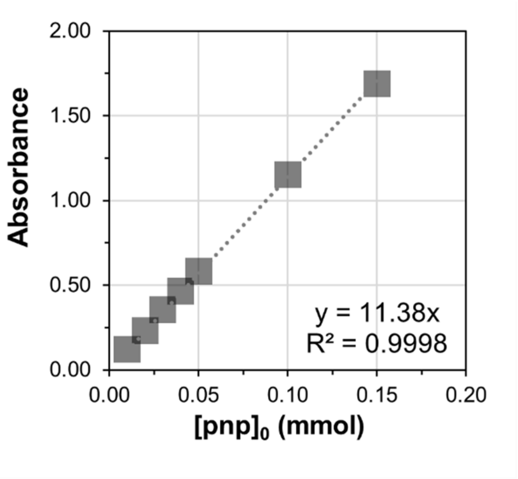

Figure S1. Calibration curve used for determination of the kinetic profile of the ALP-catalyzed reaction, measured from the absorbance of pnp solutions prepared over a relevant range of concentrations.

Figure S2

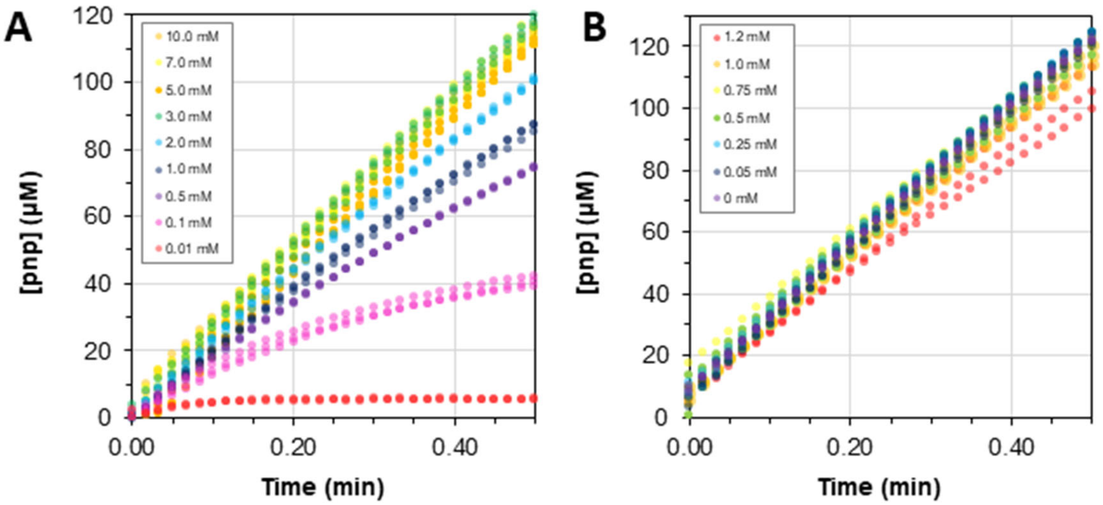

Figure S2. (A) Initial enzymatic activity measured with respect to the amount of pnpp introduced, $[pnpp]_0$, and (B) the amount of fluoride introduced, $[F^-]_0$, when $[pnpp]_0 = 7.0$ mM.

Figure S3

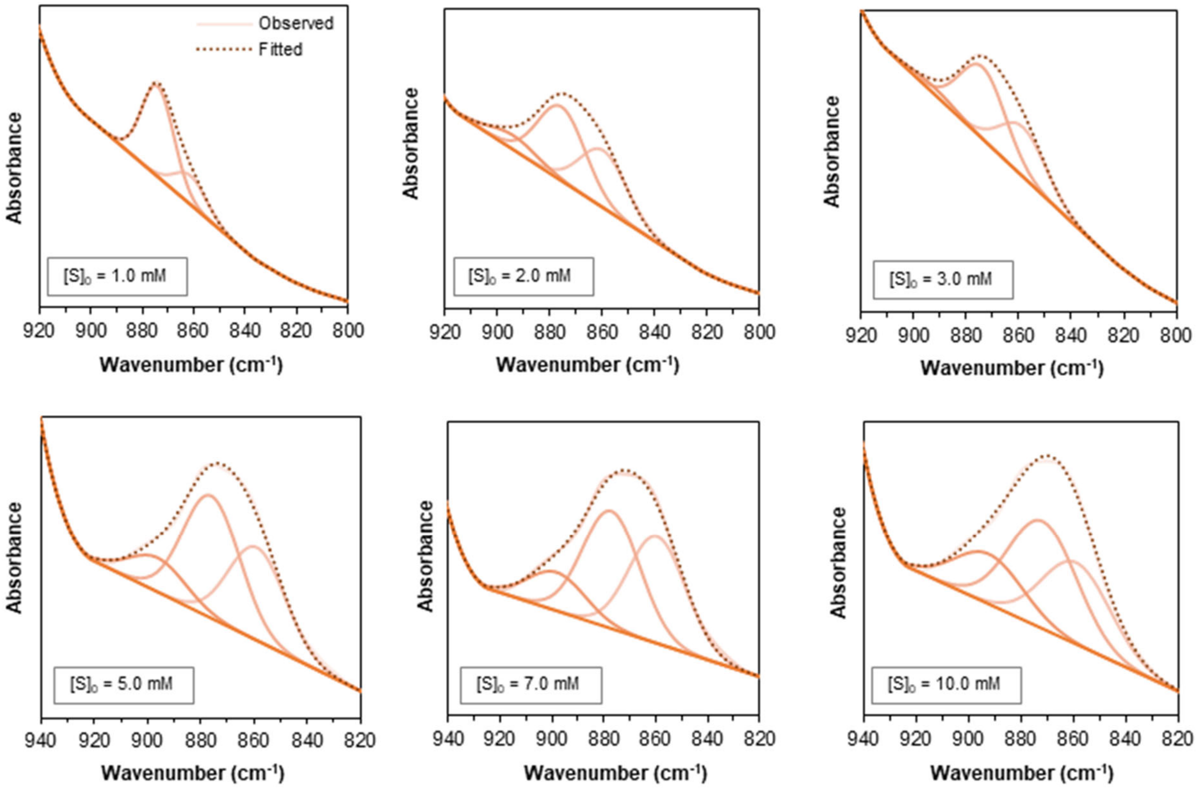

Figure S3. Curve-fitting of the ν2 CO_3^{2-} bands of selected infrared spectra for substituted hydroxyapatite prepared via enzyme-assisted mineralization with variable $[S]_0$.

The ν2 CO_3^{2-} region is fitted using either (A,B) two or (C) three components, the assignment of which were guided by second-derivative spectra and informed by consideration of the literature (See Table S1). A component at approximately 894 cm^{-1} is assigned to Type A carbonate substitution, which corresponds to the substitution of OH^- by CO_3^{2-} ions in the *c*-axis channel of the lattice; a component at approximately 874 cm^{-1} is assigned to Type B carbonate substitution, which refers to its substitution of a tetrahedral PO_4^{3-} ; a component around 858 cm^{-1} is assigned to labile carbonate, .i.e. CO_3^{2-} ions localized at the surface without being incorporated into the lattice.

Figure S4

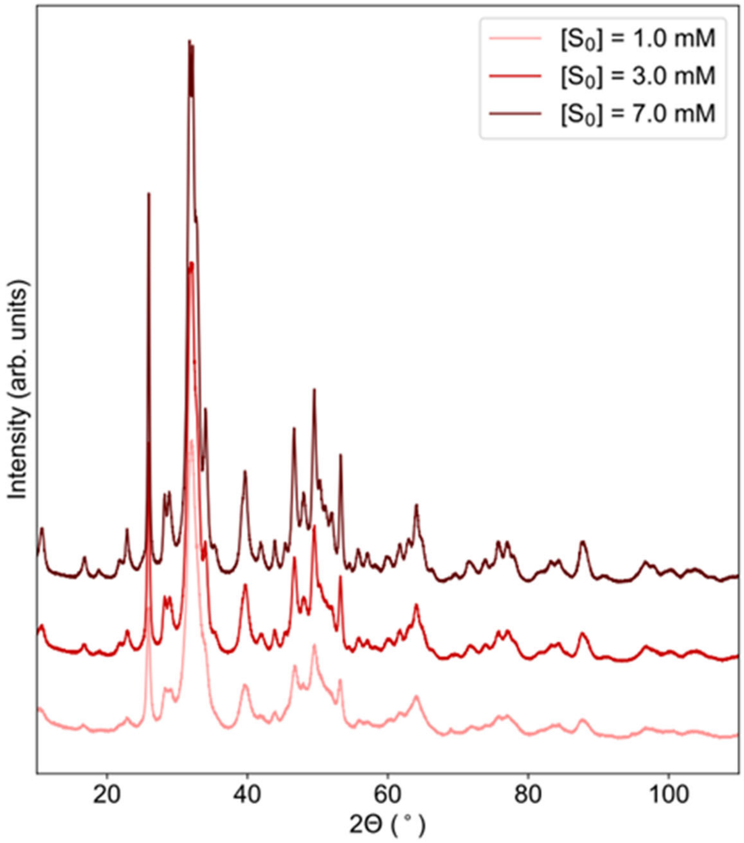

Figure S4. X-ray powder diffraction patterns for substituted hydroxyapatites synthesized via chemical precipitation at 37°C, pH 7.4 using variable concentrations of P_i.

Figure S5

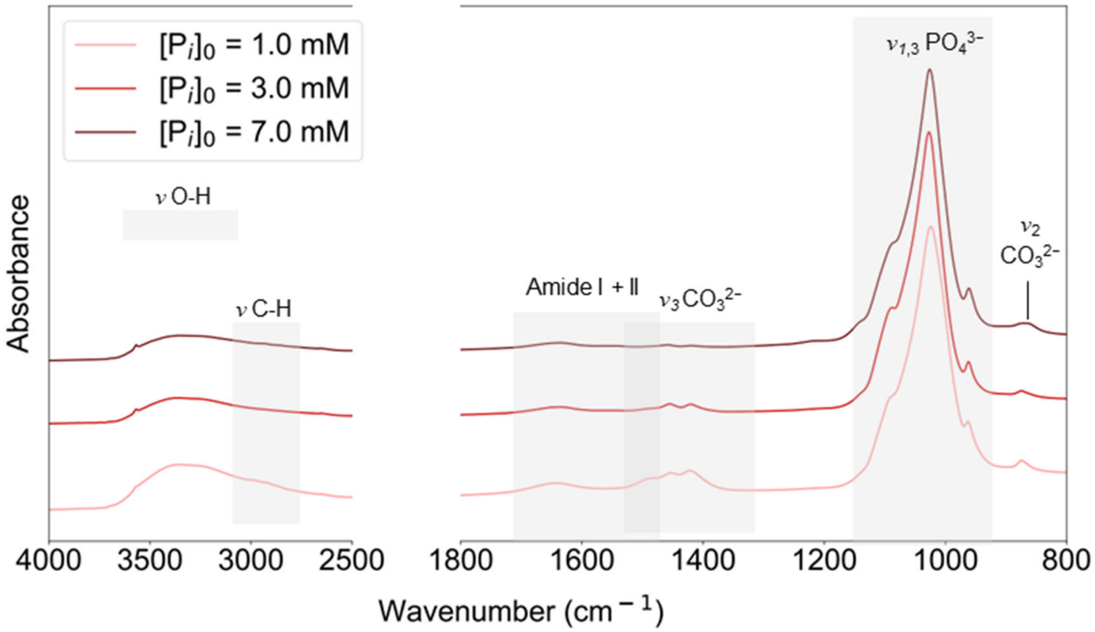

Figure S5. Selected ATR-FTIR spectra of substituted hydroxyapatite synthesized by direct precipitation in solution using a range of initial concentrations of Na_2HPO_4.

Figure S6

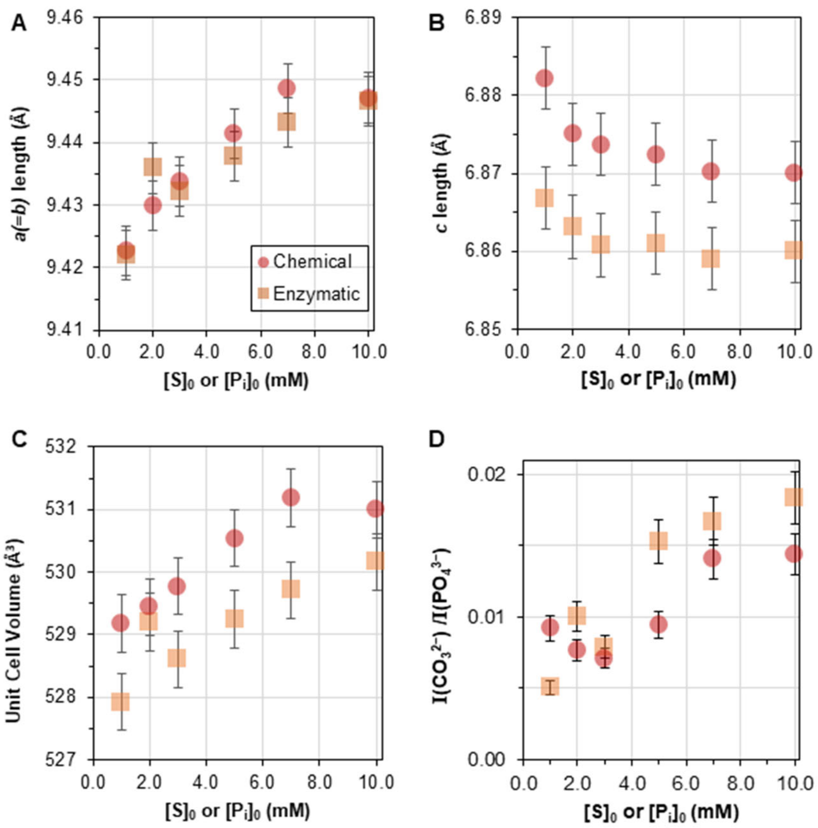

Figure S6. Comparative plots for unit cell dimensions of substituted HAP synthesized by direct precipitation ("chemical," red) compared to homogeneous enzymatic catalysis using ALP ("enzymatic," orange squares). (A,B) Lengths of unit cell parameters were determined by Rietveld refinement of X-ray powder diffraction data (C) unit cell volume calculated from the lattice parameters, and (D) carbonate-to-phosphate ratio, $I(CO_3^{2-})/I(PO_4^{3-})$, determined from IR spectra.

Overall, *a(=b)* lattice parameters measured for chemically synthesized mineral trend similarly with respect to $[P_i]$ available during synthesis compared to enzymatically synthesized mineral with respect to $[S]_0$ used (Figure S5A). The *c* lattice parameters also appear affected, showing a slight decrease with increasing $[S]_0$ in Figure S5B. Unit cell volume shows a similar trend to the enzyme-assisted mineralization but with increased volumes for the chemical synthesis (Figure S5C), but then plateau at higher concentrations of supplied phosphate (7, 10 mM). Carbonate-to-phosphate ratio showed a consistent trend at higher values of $[P_i]_0$ but was inconsistent at $[P_i]_0 < 3.0$ mM.

Figure S7

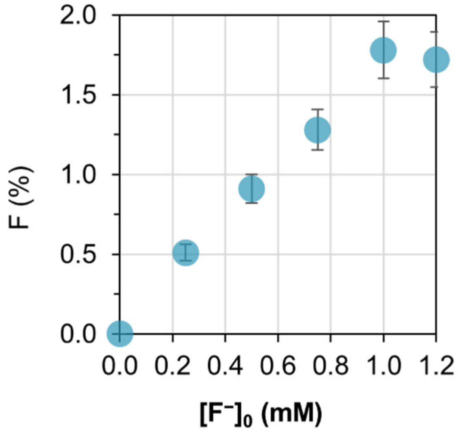

Figure S7. Plot of the molar concentration of fluoride measured by XPS (mole fraction (%) computed over the sum of all elements except hydrogen) on CaP solids mineralized using enzyme-assisted mineralization method versus the concentration of fluoride $[F^-]_0$ initially added in the mineralization solution.

Table S1

Table of crystallographic lattice parameters determined by Rietveld refinement of X-ray powder diffraction data for samples synthesized by enzyme-assisted mineralization with and without fluoride.

Enzyme-assisted Mineralization					
**$[S]_0$** (mM)	***a(=b)*** (Å)	***c*** (Å)	**$[F^-]_0$**[Ɨ] (mM)	***a(=b)*** (Å)	***c*** (Å)
1	9.422(4)	6.867(1)	0	9.452(4)	6.865(1)
2	9.436(4)	6.863(1)	0.25	9.430(4)	6.869(1)
3	9.432(4)	6.861(1)	0.5	9.416(4)	6.871(1)
5	9.438(4)	6.861(1)	0.75	9.400(4)	6.873(1)
7	9.443(4)	6.859(1)	1	9.390(4)	6.875(1)
10	9.447(4)	6.860(1)	1.2	9.389(4)	6.875(1)

[Ɨ] *$[S]_0$ = 7mM*

Table S1. Table of crystallographic lattice parameters determined by Rietveld refinement of X-ray powder diffraction data for samples synthesized by enzymatic catalysis with and without fluoride.

Table S2

Table of IR absorption band assignments:

Wavenumber (cm^{-1})	Assignments
3200-3565	*v* O-H
2800-3100	*v* C-H
1651	Amide I
1540	Amide II
1137-1138	HPO_4^{2-}
1099-1100, 1062-1063, 1027-1028, 999	v_3 PO_4^{3-} asymmetric stretching mode
960-963	v_1 PO_4^{3-} symmetric stretching mode
840-890	v_2 CO_3^{2-}
894, 899	Type A CO_3^{2-}
870, 874	Type B CO_3^{2-}
855, 858	labile CO_3^{2-}

Table S2. Table of IR absorption band assignments.

Table S3

Table of crystallographic lattice parameters determined by Rietveld refinement of X-ray powder diffraction data for samples synthesized by chemical mineralization via direct precipitation in solution.

Chemical Mineralization		
**$[P_i]_0$** (mM)	***a(=b)*** (Å)	***c*** (Å)
1	9.423(4)	6.882(1)
2	9.430(4)	6.875(1)
3	9.434(4)	6.874(1)
5	9.441(4)	6.872(1)
7	9.449(4)	6.870(1)
10	9.447(4)	6.870(1)

Table S3. Table of crystallographic lattice parameters determined by Rietveld refinement of X-ray powder diffraction data for samples synthesized by chemical mineralization via direct precipitation in solution

Method S1

Mineralization procedure for chemical mineralization:

Mineralization by direct precipitation ("chemical" method) was performed using Na_2HPO_4 and anhydrous $CaCl_2$. Na_2HPO_4 was solubilized in 10 mM Tris buffer, pH 7.4 as necessary for final solution target concentrations ($[P_i]_0$ = 1.0, 2.0, 3.0, 5.0, 7.0, and 10.0 mM) and brought to near experimental volume, then 1.0 M $CaCl_2$ stock solution (prepared in 10.0 mM Tris buffer, pH 7.4) was added to achieve a final concentration of 11.4 mM in the total volume. Solutions were incubated at 37°C with gentle agitation on a rocker plate for 48 hours. After incubation, solutions were centrifuged and supernatant was decanted. The remaining pellets were washed with equivalent volumes of MilliQ water, centrifuged, and decanted thrice, then dried at 60°C overnight (16 hours).
